# Supplementary material for: Nitrogen Fixation Activity and Genome Analysis of a Moderately Haloalkaliphilic Anoxygenic Phototrophic Bacterium Rhodovulum tesquicola
Source: Microorganisms. 2022 Aug 9;10(8):1615. doi: 10.3390/microorganisms10081615 (PMC9412634; doi:10.3390/microorganisms10081615)
Supplement: Supplementary file 1 [file Figure S1.pdf › Figure S1.pdf]

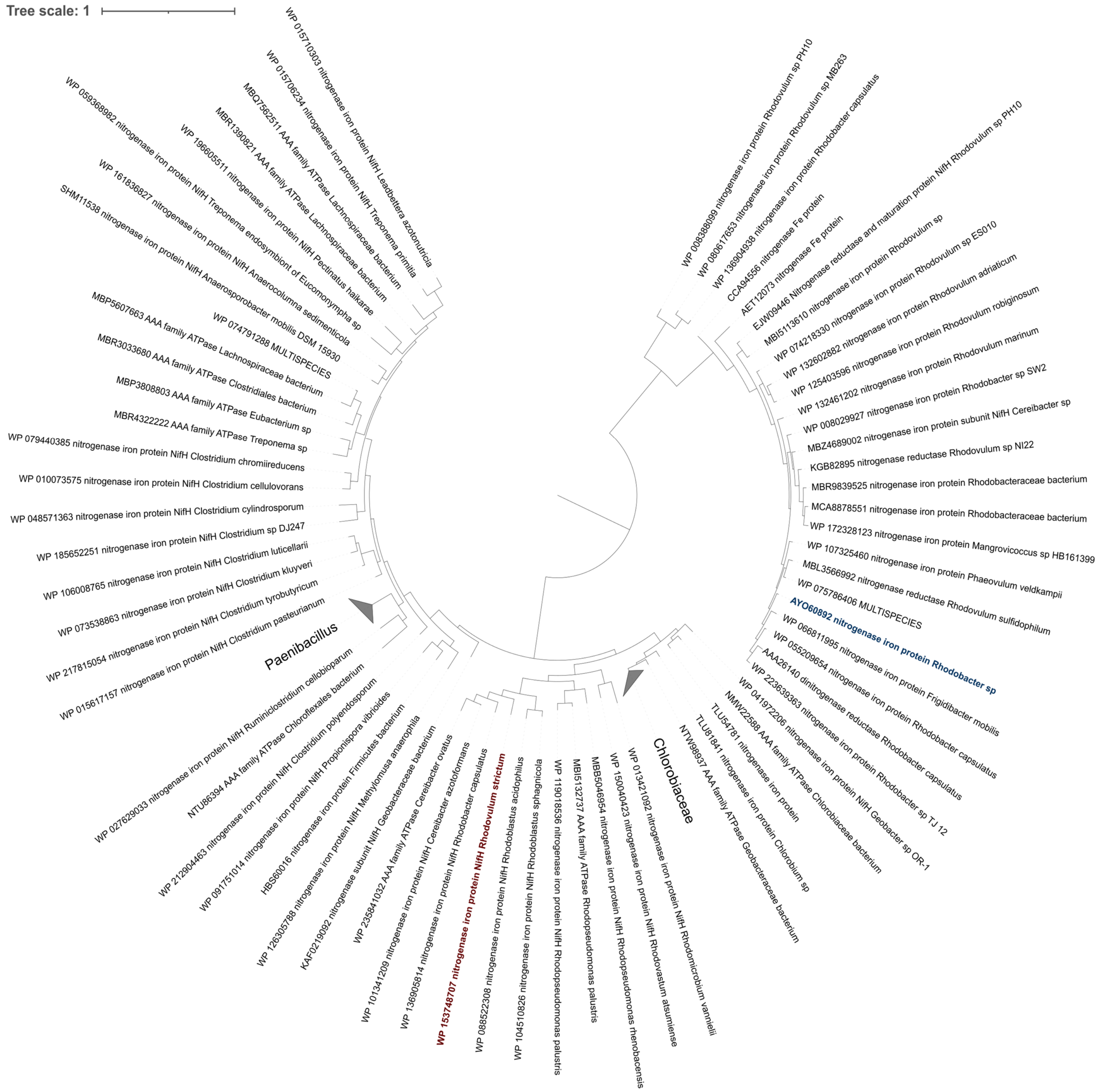

Figure S1. Phylogenetic tree of *nifH* gene across *Bacteria*. The tree includes *nifH* homologues missing in *R. steppense* and *R. tesquicola* (blue) and appearing in the three strains (red). The tree is scaled by evolutionary distance (count of substitutions per site) and rooted at the midpoint.
